# Low-load resistance training during step-reduction attenuates declines in muscle mass and strength and enhances anabolic sensitivity in older men

**DOI:** 10.14814/phy2.12493

**Published:** 2015-08-11

**Authors:** Michaela C Devries, Leigh Breen, Mark Von Allmen, Maureen J MacDonald, Daniel R Moore, Elizabeth A Offord, Marie-Noëlle Horcajada, Denis Breuillé, Stuart M Phillips

**Affiliations:** 1 Department of Kinesiology, McMaster UniversityHamilton, Canada; 2 School of Sport, Exercise and Rehabilitation Sciences, University of BirminghamBirmingham, UK; 3 Faculty of Kinesiology & Physical Education, University of TorontoToronto, Canada; 4 Nestlé Research Center, Nestec LtdLausanne, Switzerland

**Keywords:** Anabolic resistance, resistance training, sarcopenia, step-reduction

## Abstract

Step-reduction (SR) in older adults results in muscle atrophy and an attenuated rise in postprandial muscle protein synthesis (MPS): anabolic resistance. Knowing that resistance exercise (RT) can enhance MPS, we examined whether RT could enhance MPS following 2 weeks of SR. In addition, as we postulated that SR may impair feeding-induced vasodilation limiting nutrient delivery to muscle, we also examined whether citrulline (CIT), as an arginine and nitric oxide precursor, could attenuate muscle anabolic resistance accompanying SR. We used a unilateral leg model to compare older subjects’ who had undergone SR to a loaded condition of SR plus RT (SR + RT). Thirty older men (70 ± 1 years) underwent 14 days of SR (<1500 steps/day) with supplementation of either 5 g/day CIT or glycine placebo. Throughout SR, subjects performed unilateral low-load RT thrice weekly. We assessed muscle protein synthesis in the postabsorptive and postprandial state (20 g whey isolate plus 15 g glycine or as micellar-whey with 5 g CIT or 15 g glycine, *n* = 10/group). As MPS was similar after ingestion of either whey isolate, micellar-whey, or micellar-whey + CIT data related to different dietary groups were collapsed to compare SR and SR + RT legs. Subjects’ daily steps were reduced by 80 ± 2% during SR (*P* < 0.001) compared with baseline. Leg fat-free mass decreased with SR (−124 ± 61 g) and increased in the SR + RT (+126 ± 68 g; *P* = 0.003). Myofibrillar FSR was lower (*P* < 0.0001) in the SR as compared with the SR + RT leg in the postabsorptive (0.026 ± 0.001%/h vs. 0.045 ± 0.001%/h) and postprandial states (0.055 ± 0.002%/h vs. 0.115 ± 0.003%/h). We conclude that low-load RT, but not supplementation with CIT, can attenuate the deleterious effects of SR in aging muscle.

## Introduction

Sarcopenic muscle loss occurs at ∼0.8% per year beginning at around the 5th decade of life and arises, at least in part, from alterations in muscle protein turnover, such that a net negative protein balance prevails (Phillips 2009). An “anabolic resistance” of protein metabolism in aging, which is embodied as an attenuated protein synthetic response to protein ingestion, is predominantly influenced by the contractile activity of skeletal muscle. For example, we reported 2 weeks of reduced habitual activity (i.e., daily step count) induces anabolic resistance to protein feeding in otherwise healthy older adults (Breen et al. 2013). In contrast, resistance exercise is known to enhance muscle mass and can increase the sensitivity of skeletal muscle to dietary amino acids (Burd et al. 2011b).

Skeletal muscle disuse in older adults transiently accelerates muscle mass and strength losses and likely involve incomplete recovery (Suetta et al. 2009; Hvid et al. 2010, 2014). Muscular disuse results in a reduction in postabsorptive (Gibson et al. 1987; Glover et al. 2008) and postprandial (Glover et al. 2008; Wall et al. 2013) rates of myofibrillar protein synthesis (myoPS) and mixed MPS. Even modest reductions in muscular loading, via step-reduction (SR; <1500 steps/day for 2 weeks), induced muscle atrophy, and reduced insulin sensitivity in young (Krogh-Madsen et al. 2010) and older adults (Breen et al. 2013). In addition, SR reduced the postprandial myoPS response in elderly subjects (Breen et al. 2013), similar to that seen with complete immobilization (Glover et al. 2008; Wall et al. 2013), which we hypothesized was partially responsible for the atrophy observed during SR (Breen et al. 2013). As a countermeasure to disuse atrophy, resistance training (RT) enhances the anabolic sensitivity of muscle (Burd et al. 2010; West et al. 2012) and induces hypertrophy and strength gains in young and older individuals (Mitchell et al. 2012; Leenders et al. 2013). Unsurprisingly, higher load/volume RT during disuse is an effective countermeasure to atrophy and strength loss (for review see [Glover and Phillips 2010]); however, even at low volume RT attenuates disuse atrophy in young men (Oates et al. 2010), but the effects of such low volumes of work in aged persons during disuse/unloading are unknown.

Muscular disuse might impair feeding-induced vascular dilation and reduce nutrient delivery to skeletal muscle (Chadderdon et al. 2012) contributing to reduced postprandial MPS (Glover et al. 2008; Wall et al. 2013). A decline in nitric oxide (NO) production with disuse and aging may be involved in this impaired vasodilation (Casey et al. 2013). Citrulline (CIT), as an arginine precursor, ingestion could offset the reduction in the vascular impairments seen during disuse (Dai et al. 2013) and thus may offset disuse-induced vascular impairment and atrophy. Ingestion of CIT has also been shown to stimulate fasted MPS in young persons on a low-protein diet (Jourdan et al. 2014) but has no effect on postprandial myoPS in healthy older adults (Churchward-Venne et al. 2014). Nonetheless, animal models of chronic (i.e., ≥1 week) CIT ingestion have shown benefits (Osowska et al. 2006; Ventura et al. 2013); thus, we aimed to determine if prolonged administration of CIT would be beneficial in older persons.

The primary objective of this study was to determine whether low-load RT, previously shown to be effective in inducing hypertrophy in young men (Mitchell et al. 2012), with or without chronic supplementation of CIT, would enhance myoPS following a period of SR in older men. Furthermore, the secondary objectives of this study were to determine whether low-load RT and/or CIT supplementation would attenuate SR-induced muscular atrophy and strength losses. We hypothesized that low-load RT performed during SR would enhance the anabolic sensitivity of muscle to a protein-containing beverage resulting in a greater postprandial myoPS rate. Furthermore, we hypothesized that lower intensity RT would prevent decreases in muscle mass and strength during SR. In addition, we hypothesized that supplementation with CIT would further enhance the muscle anabolic effects of resistance training and independently attenuate losses in muscle mass and strength through restoration of anabolic sensitivity in the SR limb. We also investigated if a micellar form of whey protein (Schmitt et al. 2007) was similar in its ability to stimulate MPS as isolated whey protein.

## Methods

### Participants

Thirty healthy, elderly men (70 ± 1 years, 1.8 ± 0.1 m, 84.0 ± 2.6 kg; mean ± SEM) were recruited through advertisements in local newspapers for this study. This study was approved by the Hamilton Integrated Research Ethics Board (REB 11-267) and met all standards for the use of human subjects by the Canadian Tri-Council Policy on the ethical use of human subjects in research (http://www.pre.ethics.gc.ca/pdf/eng/tcps2/TCPS_2_FINAL_Web.pdf). Each participant was informed of the purpose of the study, the experimental procedures, and the potential risks prior to providing written consent. This study was registered at clinicaltrials.gov. Subjects were eligible to participate in the study if they were generally healthy men between the ages of 65 and 80 years, nontobacco users, who habitually completed ≥3500 steps per day (as assessed by a pedometer prior to commencing the trial). Subjects were excluded if they had cardiac, pulmonary, liver, or kidney abnormalities, uncontrolled hypertension, rheumatoid arthritis, diabetes, metabolic disorders, progressive degenerative neurological disease (i.e., Parkinson’s disease, multiple sclerosis, ALS), had taken any medications that would affect protein metabolism (i.e., corticosteroids, NSAIDs).

### General design

**Figure 1:**
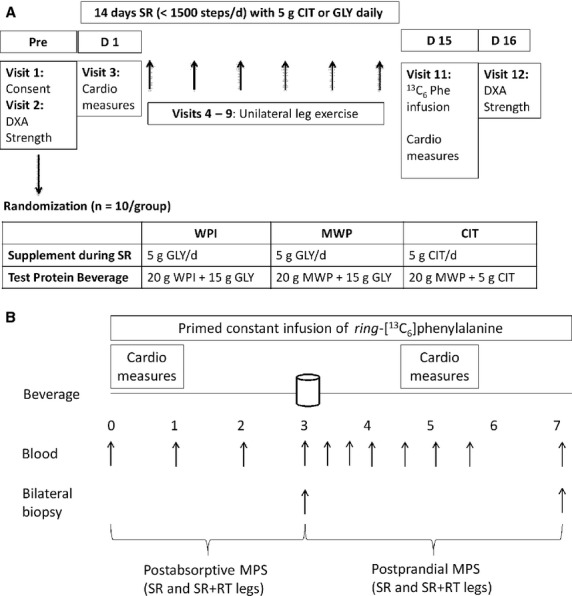
Study schematic outlining (A) the step-reduction protocol, and (B) the infusion protocol. CIT, citrulline; D, day; GLY, glycine; MWP, micellar whey protein; Phe, phenylalanine; SR, step-reduction; SR+RT, step-reduction plus resistance training; WPI, whey protein isolate.

Participants underwent a 2-week reduced step count intervention combined with a unilateral leg resistance exercise protocol to assess whether RT performed during SR, with or without CIT supplementation, would enhance the anabolic sensitivity of skeletal muscle to a mixed macronutrient protein beverage (Fig.1). We also measured changes in muscle mass, muscle function, and vascular health of both legs before and after the 2-week intervention to ascertain the effect of reduced activity and the protective effect of minimal, low-intensity exercise on these outcomes.

All subjects received an oral dietary product containing 30 g carbohydrate, 8.5 g fat, 20 g protein, and 5 g of free amino acids which correspond to a total of 272 kcal of energy. Subjects were randomized to one of three groups (*n* = 10/group) differentiated by the type of protein and free amino acids included in the product: 5 g glycine/day during step-reduction and 20 g isolated whey protein (IWP) + 15 g glycine on the infusion trial day; 5 g glycine/day during step-reduction and 20 g micellar-whey protein (MWP [Bovetto et al. 2008]) + 15 g glycine on the infusion trial day; and CIT – 5 g citrulline/day during step-reduction and 20 g MWP + 5 g citrulline on the infusion trial day. Given the ability of NO-mediated microvascular perfusion to enhance postprandial MPS (Timmerman et al. 2010, 2012), the dose of CIT in the present study was selected to be greater (i.e., 5 g vs. 3 g) than that previously reported to elicit a marked increase in the concentrations of plasma arginine and metabolic by-products of NO-production, the former of which was associated with improvements in flow-mediated dilation (Bode-Böger et al. 1998). Glycine was matched to CIT by weight (5 g) during the step-reduction period (to reduce the number of pills to be taken) and by total nitrogen (15 g) during the measure of MPS.

### Step-reduction and resistance training intervention

Prior to 14 days of SR, subjects underwent testing to assess habitual physical activity and dietary intake, body composition, strength, and vascular function. During the 14-day SR period, subjects were instructed to reduce their daily step-count to ensure they completed no more than 1500 steps per day. Subjects monitored their own step count by wearing a pedometer (Accustep 400; Accusplit, Pleasanton, CA) and recorded their daily steps each day of the intervention before bed. In addition, subjects wore an armband accelerometer (BodyMedia, Pittsburg, PA) that verified the pedometer-derived step-counts, and also provided information pertaining to physical activity intensity and daily energy expenditure. The step-count data from the armband accelerometer were inaccessible to the subject and were used as an internal validation for the subjects’ reported step counts to ensure compliance. Interdevice values differed by <10% throughout the intervention.

Throughout the SR period subjects reported to the laboratory on six occasions (three sessions/week) to undergo unilateral resistance exercise (leg press and leg extension) using compressed air resistance strength machines (HUR Health and Fitness Equipment; Northbrook, IL). Subjects were collected upon arrival to the laboratory and transported by wheelchair to the testing center where they completed three sets of each exercise at 30% of their 1-RM until volitional fatigue and/or they were unable to complete the full range of motion required for the exercise. Exercised legs (SR + RT) were randomized according to dominance. During the exercise session, the inactive leg (SR) was left in a neutral position with no resistance so as to prevent loading of the knee extensors. Subjects were also instructed to relax the muscles in their contralateral, nonexercised, inactive leg.

Upon completion of the SR protocol, subjects repeated all preliminary measures and also underwent stable isotope infusion of L-[*ring*-^13^C_6_]phenylalanine to determine the rates of muscle protein synthesis in the SR and SR + RT legs. The study is summarized schematically in Figure1A.

### Body composition and leg muscle mass

Subjects reported to the McMaster University Medical Centre 3 days prior to beginning the step-reduction intervention, as well as after the intervention (day 15; Fig.1A), to undergo assessment of their body composition (dual-energy X-ray absorptiometry (DXA) scan; QDR-4500A, Software Version 12.31; Hologic Inc., Massachusetts, USA). Body scans were used to determine body fat percentage, the total body fat mass (FM), individual leg FM, the total fat-free mass (FFM), arm FFM, individual leg FFM, and abdominal FFM. Skeletal muscle mass (SMM) was calculated using the predictive equation for lower limb SMM defined by Shih et al. (Shih et al. 2000).

### Strength testing

Maximal isometric torque and isotonic strength (single lift maximal voluntary strength – 1RM) of the knee extensors were determined prior to and following the step-reduction protocol in both the SR and SR + RT legs. Isometric knee extensor peak torque was determined during a maximal voluntary contraction (MVC) using a dynanomometer (Biodex System 3; Shirley, NY). Prior to testing, subjects were familiarized with the nature of the dynamometer testing protocol by undergoing at least one maximal contraction on each leg individually before proceeding with the test. Each unilateral test consisted of three isometric MVC of the knee extensors at an angle of 60° from the neutral 90° resting position. Each extension lasted five-seconds and was separated by 60 sec of rest at the neutral position. The highest recorded torque from each leg was taken as the highest peak isometric MVC. All settings were recorded to replicate conditions on both testing occasions. Isotonic strength of the knee extensors was determined by determining 3–5 repetition maximum strength for leg press and knee extension using compressed air resistance machines (HUR Health and Fitness Equipment, Northbrook, IL) that allow 1 kg increments in resistance. Prior to testing, subjects were familiarized with the knee extension and leg press machines by performing a single set of nonfatiguing weight for at least 10 repetitions. Subjects rested for 2 min and then started the 3–5 RM testing. Unilateral 3–5 RM values were determined for both leg press and leg extension within four attempts with each attempt separated by 2 min and 1RM values were estimate to determine the appropriate 30% of 1RM load for resistance training.

### Infusion protocol

Upon completion of the step-reduction protocol, subjects reported to the laboratory to undergo an assessment of fasted and fed MPS (Fig.1B). Subjects reported to the laboratory in the fasted state and a catheter was inserted into an antecubital vein of each arm. One arm was wrapped in a 45°C heating blanket to allow for “arterialization” of venous blood. After a baseline blood sample had been taken, a primed (2 *μ*mol kg^−1^), constant (0.05 *μ*mol kg^−1^ min) infusion of L-[*ring*-^13^C_6_]phenylalanine was initiated (Cambridge Isotopes, Tewksbury, MA). Muscle biopsies were taken to measure MPS in fasting conditions under local anesthetic (1% xylocaine) from the *vastus lateralis* of the SR and SR + RT legs after 150 min of infusion using a custom-modified 5 mm Bergström needle with manual suction. Muscle biopsies were dissected free of visible fat and connective tissue and rapidly frozen in liquid nitrogen and stored at −86°C until subsequent analysis. Immediately following the muscle biopsy, subjects ingested their assigned study beverage (described above). All drinks were 400 mL and were enriched to 5% with [*ring*-^13^C_6_]phenylalanine, based on measurement of the protein phenylalanine content, to minimize disturbances in isotopic steady state upon ingestion and were isocaloric and isonitrogenous. Arterialized blood samples were taken as outlined in Figure1B and were processed as previously described (Moore et al. 2009). At 240 min after consumption of the drink, a second muscle biopsy was taken from the SR and SR + RT legs to calculate muscle protein fractional synthetic rate in fed conditions (see below).

### Vascular assessment and analysis

Given the role of CIT as an arginine and NO precursor, we assessed the effect of acute and chronic CIT supplementation on vascular parameters including blood pressure, femoral artery diameter, and femoral blood flow. Thus, prior to commencing the step-reduction and on the acute infusion trial day, prior to (fasted) and 90 min following drink consumption (fed), vascular measurements were made. Continuous measurements of heart rate via single lead electrocardiograph (ECG) (ML123, ADInstruments Inc. Colorado Springs, CO) and blood pressure (BP) measurements via an automated applanation tonometer with and without oscillometric cuff calibration (Nexfin monitor, Model 1, BMEYE B.V., Amsterdam, The Netherlands or Finometer Midi, Model 2, & Finapres Medical Systems, Amsterdam, The Netherlands) were made. Immediately thereafter assessment of right and left common femoral artery diameter (∼2–3 cm proximal to the bifurcation of the superficial and profundus segments) and blood flow was made using a Vivid q BT 10 (General Electric Healthcare, Milwaukee, WI) echocardiography console and linear array transducer (12L-RS) as previously described (Churchward-Venne et al. 2014).

### Blood analysis

Plasma [^13^C_6_] phenylalanine enrichments were determined by gas chromatography–mass spectrometry (GCMS; GC: 6890N, MS: 5973; Hewlett-Packard, Palo Alto, CA) as previously described (Glover et al. 2008). Blood total (except arginine and citrulline), essential (EAA), and branched chain (BCAA) amino acid and leucine concentrations were determined using the EZfaast amino acid analysis kit (Phenomenex, Torrance, CA) and GCMS (GC: 6890N, MS: 5973; Hewlett-Packard, Palo Alto, CA) as per the manufacture instructions. Plasma citrulline and arginine concentrations were analyzed using high-performance liquid chromatography (HPLC), as described previously (Wilkinson et al. 2007). Plasma glucose and insulin were measured as described previously (Glover et al. 2008).

### Muscle analyses

Muscle intracellular (IC) free amino acids were extracted from a 10–15 mg piece of wet muscle over ice using ice-cold 0.6 mol/L perchloric acid (PCA) and purified as previously described (Di Donato et al. 2014). Purified free amino acids were passed over an ion exchange resin (Dowex 50W8-200 resin) and converted to their heptafluorobutyrate (HFB) derivatives and analyzed for [*ring*-^13^C_6_] phenylalanine enrichment using GCMS (GCMS; GC: 7890N, MS: 5973; Hewlett-Packard, Palo Alto, CA) as previously described (Di Donato et al. 2014).

A separate piece of muscle tissue was homogenized to determine myofibrillar and sarcoplasmic enrichments. Muscle samples (∼30–50 mg) were homogenized on ice in buffer (10 *μ*L/mg 25 mmol/L Tris 0.5% vol/vol Triton X-100 and protease/phosphatase inhibitor cocktail tablets [Complete Protease inhibitor Mini-Tabs, Roche, Indianapolis, IN and PhosSTOP, Roche Applied Science, Mannhein, Germany]) and centrifuged at 15,000 *g* for 10 min at 4°C. The supernatant was used to determine sarcoplasmic protein enrichments, whereas the pellet was used to determine myofibrillar protein enrichments. The myofibrillar protein pellet was solubilized and centrifuged as previously described (Burd et al. 2011a) and the supernatant containing the myofibrillar proteins was collected. Sarcoplasmic and myofibrillar proteins were isolated and analyzed as previously described using gas chromatography combustion-isotope ratio mass spectrometry (Burd et al. 2011a). The fractional protein synthetic rate of the myofibrillar and sarcoplasmic protein fractions was calculated using the precursor-product equation as described (Burd et al. 2011a; Breen et al. 2013) and using a blood sample from “tracer naïve” subjects as the baseline enrichment for calculation of fasting MPS, an approach that has been previously validated in our laboratory (Burd et al. 2011a).

### Statistics

Statistical analyses were completed using SPSS (version 22.0, Chicago, IL, USA). Analyses of variance (ANOVA) were used as appropriate for variables with a single level, but multiple groups (one-way ANOVA), two levels (two-way ANOVA), or three levels (three-way ANOVA). Both protein fractions, myoPS and sarcoplasmic MPS (sarcPS), were initially assessed with a three-way mixed model ANOVA with beverage (CIT, IWP, MWP) as a between factor and time (fasted, fed) and leg (SR, SR + RT) as within factors. As myofibrillar FSR was the primary outcome variable of interest when no beverage-specific differences or interactions were seen with this variable, data were collapsed across groups and a two-way mixed model ANOVA was conducted to determine the effect of time and leg for all study outcomes. The effects of the intervention on body weight and composition, physical activity, and daily energy expenditure were determined using paired *t*-tests. Tukey’s post hoc test was used where appropriate to isolate specific pairwise differences. A value of *P* < 0.05 was determined to indicate a statistically significant result. Results are reported as means ± SEM unless otherwise specified.

## Results

### Subject characteristics and physical activity

There were no differences in age, weight, height, or BMI between groups at baseline and step-reduction did not alter body weight or BMI (Table1). In addition, there were no differences in average daily step count, average daily energy expenditure, average daily physical activity >3 metabolic equivalents (MET), or leg strength prior to step-reduction between groups. Two weeks of SR significantly decreased average daily steps by ∼82% (*P* < 0.0001), the amount of physical activity performed at >3 MET by ∼86% (*P* = 0.0001), and daily energy expenditure by 14% (*P* < 0.0001, Table1).

**Table 1:** Subject characteristics by group prior to and following 14 days of step-reduction in older men

	IWP		MWP		CIT	
	Pre	Post	Pre	Post	Pre	Post
Body weight (kg)	80.9 ± 2.9	81.6 ± 3.5	84.0 ± 6.2	84.2 ± 6.2	87.1 ± 4.0	87.7 ± 4.1
BMI (kg/m^2^)	26.1 ± 1.0	26.4 ± 1.1	26.2 ± 1.3	26.3 ± 1.3	27.7 ± 0.7	28.0 ± 0.7
Daily steps	7714 ± 809	1288 ± 62*	7119 ± 797	1270 ± 88*	6273 ± 980	1161 ± 107*
Daily PA > 3 METs (min/d)	75 ± 14	27 ± 6*	96 ± 33	25 ± 7*	81 ± 18	29 ± 10*
Daily EE (kcal/d)	2482 ± 156	2143 ± 123*	2657 ± 193	2136 ± 158*	2392 ± 95	2132 ± 39*

Data are means ± SEM.

#, number; >, greater than; avg, average; CIT, citrulline; d, day; EE, energy expenditure; IWP, isolated whey protein; kcal, kilocalories; METs, metabolic equivalents; min, minutes; MWP, micellar whey protein; PA, physical activity.

* Significantly different from pre, *P* < 0.001.

### Myofibrillar and sarcoplasmic protein synthesis

**Figure 2:**
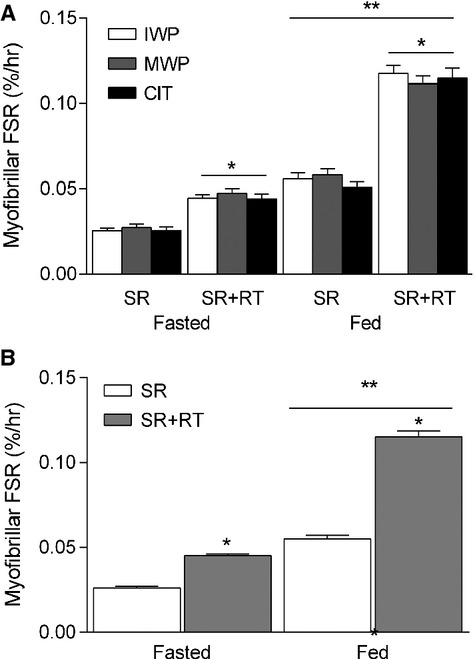
Postabsorptive and postprandial myofibrillar fractional synthetic rate (FSR; %/h) (A) by group and (B) with data collapsed in the step-reduced (SR) and step-reduced + resistance trained (SR + RT) leg following 14 days of reduced ambulation in older men. *significantly greater than SR, *P* < 0.001; **significant increase in response to feeding, *P* < 0.001). Values are means ± SEM (*n* = 30).

None of the dietary treatments induced a differential myoPS response (Fig.2A). The two forms of whey (IWP and MWP) induced a similar twofold increase in myoPS following ingestion of the products. Despite increases in plasma citrulline (CIT: 1080 ± 66 *μ*M vs. IWP and MWP: 84 ± 8 *μ*mol/L) and arginine (CIT: 190 ± 7 *μ*mol/L vs. IWP and MWP: 115 ± 4 *μ*mol/L) concentrations following CIT ingestion (both *P* < 0.0001), there was no further effect of citrulline supplementation on myoPS (Fig.2A). Since the MPS response was not different among the three dietary treatments, we opted to collapse the results of the three groups for MPS and for all other outcome variables to determine the overall effects of SR and SR + RT independent of dietary treatment. MyoPS was lower in the SR as compared with the SR + RT legs in both the postabsorptive and postprandial states (*P* < 0.0001, Fig.2B). Feeding increased myoPS in both legs; however, the increase was greater in the SR + RT leg (*P* < 0.001). Similar results were found for sarcoplasmic protein synthesis (sarcPS) whereby rates were lower in the SR limb in both the postabsorptive (SR: 0.036 ± 0.001%/h vs. SR + RT: 0.058 ± 0.002%/h) and postprandial states (SR: 0.056 ± 0.002%/h vs. SR + RT: 0.104 ± 0.003%/h) as compared with SR + RT leg (*P* < 0.001). Feeding increased sarcPS in both legs (*P* < 0.001) with a greater increase found in the SR + RT leg (SR: 0.022 ± 0.002%/h vs. 0.049 ± 0.005%/h, *P* < 0.001).

### Body composition

**Figure 3:**
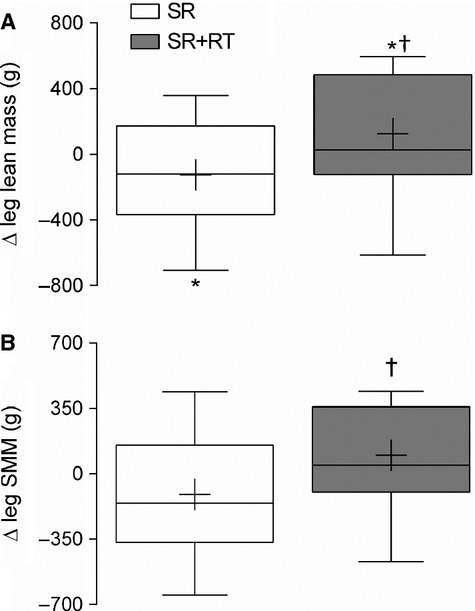
Box and whisker plots showing the change in leg (A) fat-free mass,and (B) skeletal muscle mass in the step-reduced (SR) and step-reduced + resistance trained (SR + RT) leg prior to and following 14 days of step-reduction in older men. *Significantly different from baseline, *P* < 0.01; †significantly different between limbs, *P* < 0.01. Values are medians (line), means (+), and ±95% CI (*n* = 28).

Whole-body and leg composition are summarized in Table2. There was no effect of SR on total FFM or FM, % body fat, or appendicular lean mass. SR significantly decreased leg FFM, whereas SR+RT significantly increased leg FFM such that leg FFM differed between legs (*P* = 0.003, Fig.3). Similarly, SMM also differed between legs (*P* = 0.004, Fig.3). There was no effect of SR or SR + RT on leg FM.

**Table 2:** Whole-body and leg composition prior to and following 14-days of step-reduction in older men

	Pre	Post
Total FFM (g)	56240 ± 1340	56649 ± 1400
Total FM (g)	22083 ± 1543	22016 ± 1556
Body Fat (%)	25.3 ± 1.2	25.1 ± 1.2
Appendicular lean mass (kg)	8.1 ± 0.1	8.2 ± 0.1
Leg FFM (g)		
SR	9191 ± 216	9067 ± 218*
SR + RT	9206 ± 258	9332 ± 254*^,^†
Leg SMM (g)		
SR	7086 ± 170	7013 ± 175
SR + RT	7096 ± 200	7196 ± 200‡
Leg FM (g)		
SR	3354 ± 247	3289 ± 241
SR + RT	3338 ± 239	3284 ± 239

Data are means ± SEM.

FFM, fat free mass; FM, fat mass; SMM, skeletal muscle mass.

* Different from pre, *P* = 0.003.

† Greater than SR post, *P* = 0.003.

‡ Greater than SR post, *P* = 0.004.

### Leg strength

**Figure 4:**
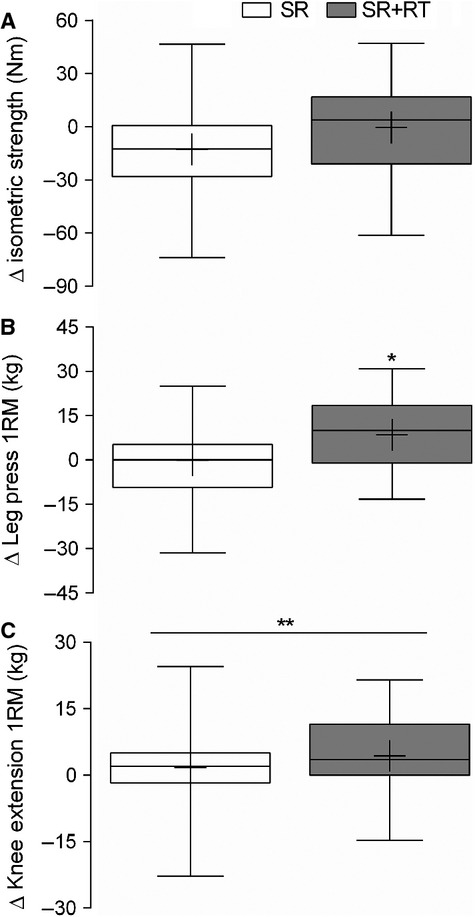
Box and whisker plots showing the change in (A) isometric knee extensor strength (*n* = 28), (B) leg press 1 RM (*n* = 26), (C) knee extensor 1 RM (*n* = 26) in the step-reduced (SR) and step-reduced + resistance trained (SR + RT) leg following 14 days of reduced ambulation in older men. *Different from baseline, *P* = 0.002; **different from baseline, *P* = 0.008. Values are medians (line), means (+), and ±95% CI.

Isometric maximal voluntary contraction of the knee extensors did not change over the course of the intervention and was not different between legs (Fig.4A). Leg press 1RM increased in the SR + RT in response to training (*P* = 0.002) and did not change in the SR leg and thus leg press 1RM was greater in the SR + RT as compared with the SR leg (Fig.4B). On the other hand, knee extension 1RM increased over the course of the intervention in both legs (*P* = 0.008, Fig.4C).

### Vascular measures

Vascular outcomes are summarized in Table3. There was no effect of SR or SR + RT on femoral artery diameter, leg blood flow, or mean arterial pressure. Heart rate was higher in the fed state as compared to the fasted state (*P* < 0.0001) with no difference between the times when leg blood flow measurements were made on each leg.

**Table 3:** Vascular measures prior to and following 14-days of step-reduction in older men

	Pre	Post – fasted	Post – fed
Femoral artery diameter (cm)			
SR	1.03 ± 0.03	1.05 ± 0.04	1.09 ± 0.03
SR + RT	1.05 ± 0.04	1.08 ± 0.03	1.11 ± 0.03
Leg blood) flow (mL/min			
SR	229 ± 32	276 ± 27	251 ± 35
SR + RT	216 ± 35	262 ± 31	221 ± 23
Leg blood flow/SMM (mL/kg min)			
SR	31 ± 5	38 ± 4	36 ± 5
SR + RT	28 ± 5	34 ± 5	30 ± 4
Leg pulse (BPM)			
SR	61 ± 2	62 ± 2	65 ± 2*
SR + RT	62 ± 2	63 ± 2	67 ± 2*
Mean arterial pressure (mmHg)	78 ± 3	83 ± 3	82 ± 3

Data are means ± SEM.

BPM, beats per minute; SMM, skeletal muscle mass.

* Greater than pre and post – fasted, *P* < 0.0001.

### Plasma glucose, insulin, and amino acids

Plasma glucose concentration transiently increased over the first 60 min after drink consumption compared with baseline values, after which, concentrations returned to baseline values by 90 min and (*P* < 0.0001, Table4). Plasma insulin was increased above baseline values at 20 min after drink consumption, peaking at 40 min after drink consumption and remaining above baseline levels beyond 120 min after drink consumption (*P* < 0.0001, Table4). Plasma EAA concentration increased above baseline by 20 min after drink consumption and remained elevated for the remainder of the trial (*P* < 0.001, Table4). Plasma BCAA concentration increased above baseline by 20 min after drink consumption and returned to baseline levels by the end of the trial (*P* < 0.0001, Table4). There was a main effect for time for plasma leucine concentration to increase above baseline within 20 min of drink consumption, peak 60 min after drink consumption, and then slowly decrease, remaining above baseline concentrations for the remainder of the trial (*P* < 0.001, Table4).

**Table 4:** Plasma glucose, insulin, ∑EAA, ∑BCAA and leucine variables after study beverage consumption

	AUCpos (AU)	Tmax (min)	Cmax
Glucose (mmol/L)	90 ± 14	44 ± 4	7.4 ± 0.2
Insulin (*μ*IU/mL)	4456 ± 571	47 ± 5	71.5 ± 8.7
∑EAA (nmol/mL)	35,327 ± 4092	89 ± 9	1079.0 ± 38.0
∑BCAA (nmol/mL)	20,089 ± 2190	94 ± 10	531.4 ± 19.8
Leucine (nmol/mL)	10,894 ± 1018	113 ± 12	189.0 ± 8.0

Data are means ± SEM.

AUC was calculated from data obtained in the 120 min following drink consumption for plasma glucose and insulin and the 240 min following drink consumption for amino acids.

∑BCAA, sum of branched chain amino acid concentrations; ∑EAA, sum of essential amino acid concentrations; AU, arbitrary units; AUCpos, area under the curve above baseline; Cmax, maximum concentration; Tmax, time at maximum concentration.

### Plasma and intracellular free phenylalanine enrichments

Plasma enrichment of ^13^C_6_ phenylalanine did not differ across time throughout the infusion protocol (*P* = 0.07). Intracellular free phenylalanine enrichments (SR fasted: 0.0516 ± 0.0005, SR + RT fasted: 0.0513 ± 0.0004, SR fed: 0.0520 ± 0.0005, SR + RT fed: 0.0517 ± 0.0004) did not differ between legs (*P* = 0.40) or across time (*P* = 0.22).

## Discussion

We found that thrice weekly sessions of low-load resistance exercise, but not CIT supplementation, offset the deleterious changes induced by 14 days of SR in older men. We report that rates of myofibrillar and sarcoplasmic protein synthesis were lower in the SR leg as compared with the SR + RT leg in both the postabsorptive and postprandial states. The finding of a reduced postprandial rise in MPS in the SR leg compared to the SR + RT leg is consistent with our previous work in this model with older adults (Breen et al. 2013), although it is possible that the difference between legs was the result of an acute exercise-induced increase in the SR + RT. We also found that leg FFM and SMM decreased in a leg that had undergone SR, but increased in the contralateral SR + RT leg. Contrary to our hypothesis, however, we did not find an effect of CIT supplementation in attenuating muscle atrophy, enhancing the muscle protein synthetic response, or altering vascular outcomes in either the SR or the SR + RT legs. Similar to our previous work, we found no effect of SR on strength (Breen et al. 2013); however, we did find that low-load, high-volume resistance exercise was able to induce increases in leg press maximal strength (1RM) in spite of concomitant SR, with no effect on isometric maximal voluntary contraction force. As expected, resting femoral artery blood flow and diameter was not different between the groups; however, contrary to our hypothesis, we did not observe acute increases in femoral artery blood flow with CIT supplementation.

Previously, we reported that 2 weeks of SR decreased MPS in the postprandial but not postabsorptive state (Breen et al. 2013). In other models of muscle disuse (i.e., bed-rest, limb immobilization), there are reduced rates of MPS in both the postabsorptive (Gibson et al. 1987; Glover et al. 2008) and postprandial states (Glover et al. 2008; Wall et al. 2013). The effectiveness of RT in maintaining MPS during disuse is quite clear (Ferrando et al. 1997). Moreover, we have demonstrated that low-load resistance exercise can enhance fasted myoPS and enhance its sensitivity to dietary protein for up to 24 h in young adults (Burd et al. 2011b). Interestingly, we found that myoPS was significantly higher in the SR + RT, as compared with the SR leg, in both the postabsorptive and postprandial states despite the last RT session occurring ∼72 h prior to the infusion protocol, suggesting that the myoPS response to RT is sustained in older adults. Importantly, the differences in myoPS between the SR and SR + RT legs seen in the current study are congruent with the differential changes in leg skeletal muscle mass we observed. However, since we did not assess pre-SR rates of myoPS, the difference in myoPS between groups in the present study may have been related to a SR-induced reduction in myoPS in the SR leg (Breen et al. 2013), and/or an RT-induced augmentation of myoPS in the SR + RT leg (Burd et al. 2011b).

Similar to our previous work (Breen et al. 2013), we found a decrease in leg lean mass following 2 weeks of SR. Interestingly, we report that there were significant gains in muscle mass and strength despite concomitant SR, with thrice weekly low-load RT. Similar effects of RT, with much heavier loading protocols, in preventing atrophy during overt disuse have been shown previously (Kawakami et al. 2001; Brooks et al. 2008). However, previously we reported that low-load RT elicits similar gains in muscle mass as well as strength in unpracticed tasks as a traditional high-load RT regime (Mitchell et al. 2012). The results from the present study demonstrate that the loading stimulus required to prevent atrophic muscle loss during disuse can be minimal. These findings are supported by those of Gibson et al. (Gibson et al. 1988), who showed minimal neuromuscular electrical stimulation (NMES) could offset immobilization-induced muscle atrophy. However, NMES may not always be as effective as RT in attenuating atrophy in older adults (Suetta et al. 2008). Given the increase in leg lean mass and strength induced by low-load RT we observed, we propose that this RT regime may be an effective tool in, for example, rehabilitation in aging persons.

As compared to our previous investigation (Breen et al. 2013), SMM decreased to a lesser extent in the SR limb in the current investigation (1.4% vs. 3.9%). Krogh-Madsen (Krogh-Madsen et al. 2010) reported a 2.7% loss of leg lean mass in young men following 2 weeks of SR. We speculate that the smaller loss of SMM in the present study may have been due to a cross education effect whereby the strength of the muscles in an untrained limb increases in response to training of the homologous muscles of the contralateral limb (for a review see [Carroll et al. 2006]). Recently, several trials have shown that the strength of an immobilized arm can be maintained when the contralateral limb undergoes resistance training during the immobilization period (Farthing et al. 2009, 2011; Magnus et al. 2010). It is, however, equivocal as to whether cross education to an immobilized limb attenuates atrophy with studies showing no effect (Farthing et al. 2011) or a relative preservation of muscle thickness (Farthing et al. 2009; Magnus et al. 2010). These findings could be important since the preservation of muscle mass and strength of an immobilized limb by training the contralateral limb has implications for rehabilitation and/or postsurgical management of individuals with unilateral limb injury as has recently been suggested (Farthing and Zehr 2014). It is also important to put our findings, seen in a two-week period, in some context of what it means in aging adults. Sarcopenic muscle mass loss is estimated at ∼0.8% per year (Phillips 2009). In the current study, the step-reduced limb lost 1.3% and the step-reduced and resistance exercised limb gained 1.4% lean mass, respectively in only 2 weeks. The loss in lean mass that occurred over 2 weeks represents an almost twofold greater loss than that typically seen in 1 year with aging. Similarly, the fact that such a minimal amount of resistance exercise (thrice-weekly low-load, high volume resistance exercise) during step-reduction was not only able to completely prevent, but actually increase lean mass is, we propose, of clinical relevance. Our work serves, we submit, to illustrate just how important physical inactivity is in older adults who may struggle to regain muscle that they have lost due to inactivity (Suetta et al. 2009).

Feeding induced microvascular blood flow is impaired in older adults (Mitchell et al. 2013) and may contribute to the reduced anabolic response to feeding in this population (Moore et al. 2015). It has been shown that pharmacological NO-administration (Timmerman et al. 2010), walking (Timmerman et al. 2012), and resistance exercise (Phillips et al. 2015) can improve microvascular perfusion, amino acid delivery, and subsequently postprandial mixed MPS in older individuals. Since NO synthase expression and/or activity may be compromised with age (Casey et al. 2013), we speculated that CIT may, by increasing NO production [to enhance microvascular flow (Dai et al. 2013)], increase amino acid delivery to the muscle stimulating myoPS and that this effect would be most pronounced in the SR leg. Contrary to our hypothesis, we did not find an effect of chronic or acute CIT ingestion in increasing conduit artery blood flow or enhancing myoPS with feeding in either the SR or SR + RT limbs. Our findings are similar to our previous work showing no effect of acute CIT supplementation (10 g) on microvascular flow or the MPS response to low dose (15 g) protein ingestion in older adults (Churchward-Venne et al. 2014). In contrast, Jourdan et al. found (Jourdan et al. 2014) that administration of 11–24 g of CIT over the course of 8 h increased rates of muscle protein synthesis to a greater extent than isonitrogenous nonessential amino acids with consumption of a low-protein diet (0.66 g/kg/day) for the 3 days prior to the determination of MPS. Differing doses of CIT, age of participants, and the level of protein in the diet between the current study, our previous work (Churchward-Venne et al. 2014), and that of Jourdan et al. (Jourdan et al. 2014) means the differing results are perhaps not surprising. The present dose of CIT (5 g) was slightly greater than a previous study that demonstrated a robust increase in plasma arginine concentration and markers of NO-metabolism with as little as 2 g supplementation; however, the authors suggested a clinically relevant dose of CIT to maximize arginine and NO synthesis may be as high as 10 g (Moinard et al. 2008). Nevertheless, our previous work (Churchward-Venne et al. 2014) did incorporate ingestion of a larger CIT dose (10 g) and we did not see increased microvascular or conduit artery blood flow or muscle protein synthesis in healthy older men. However, our dose of 5 g in the current study and 10 g in our previous trial resulted in a peak plasma arginine concentrations of 190 ± 7 *μ*mol/L and 248 ± 17 *μ*mol/L, respectively, which as noted by Churchward-Venne (Churchward-Venne et al. 2014), was much lower than the peak plasma concentration of 6200 *μ*mol/L that was found to increase vasodilation following intravenous arginine administration (Bode-Böger et al. 1998). As such, the dose of CIT used in this study, despite being given for 2 weeks, may not have been adequate to improve the feeding-induced vasodilatory response. In older adults, a diminished NO-induced vasodilatory response to a single-muscle contraction has been established (Casey et al. 2013). A lower NO bioavailability in older adults, at least in part, underpins this attenuated vasodilatory response (Donato et al. 2009). It has been speculated that impaired feeding-induced increases in leg blood flow may, at least in part, be responsible for the blunted MPS response to feeding in older adults (Phillips et al. 2012; Mitchell et al. 2013), however subsequent studies have failed to link improvements in leg blood flow induced by resistance exercise training (Phillips et al. 2015) or methacholine infusion (Phillips et al. 2014) to an enhanced MPS response. Taken together, the results of the current study and our earlier work (Churchward-Venne et al. 2014) suggest that CIT does not act to increase/restore microvascular circulation or MPS in healthy older men at rest, following exercise or following a period of inactivity.

In conclusion, our results support a role for low-load RT performed during SR to enhance muscle anabolic sensitivity in older adults. The greater anabolic sensitivity of the RT limb is congruent with the subsequent phenotypic changes in muscle mass and strength. Contrary to our hypothesis, chronic CIT supplementation enhanced neither femoral blood flow nor MPS in either a SR or a SR + RT limb despite dramatically increasing plasma arginine availability. Together these findings suggest a greater role for mechanical, as opposed to nutritional, stimuli to preserve muscle mass, and function during periods of reduced activity. Given the increased frequency with which older adults are exposed to short-term periods of overt or subtle muscle disuse, and the potential for incomplete recovery from such, the findings presented herein indicate that concomitant low-load RT is an appropriate strategy to offset the deleterious consequences of SR-induced muscle disuse in older men.
